# Analysis of the complete genome sequence of *Nocardia seriolae* UTF1, the causative agent of fish nocardiosis: The first reference genome sequence of the fish pathogenic *Nocardia* species

**DOI:** 10.1371/journal.pone.0173198

**Published:** 2017-03-03

**Authors:** Motoshige Yasuike, Issei Nishiki, Yuki Iwasaki, Yoji Nakamura, Atushi Fujiwara, Yoshiko Shimahara, Takashi Kamaishi, Terutoyo Yoshida, Satoshi Nagai, Takanori Kobayashi, Masaya Katoh

**Affiliations:** 1 Research Center for Bioinformatics and Biosciences, National Research Institute of Fisheries Science, Japan Fisheries Research and Education Agency, Yokohama, Kanagawa, Japan; 2 Research Center of Fish Diseases, National Research Institute of Aquaculture, Japan Fisheries Research and Education Agency, Saiki, Oita, Japan; 3 Fisheries Agency, Ministry of Agriculture, Forestry and Fisheries, Chiyoda-ku, Tokyo, Japan; 4 Faculty of Agriculture, University of Miyazaki, Miyazaki, Japan; 5 Headquarters, Japan Fisheries Research and Education Agency, Yokohama, Kanagawa, Japan; Academia Sinica, TAIWAN

## Abstract

Nocardiosis caused by *Nocardia seriolae* is one of the major threats in the aquaculture of *Seriola* species (yellowtail; *S*. *quinqueradiata*, amberjack; *S*. *dumerili* and kingfish; *S*. *lalandi*) in Japan. Here, we report the complete nucleotide genome sequence of *N*. *seriolae* UTF1, isolated from a cultured yellowtail. The genome is a circular chromosome of 8,121,733 bp with a G+C content of 68.1% that encodes 7,697 predicted proteins. In the *N*. *seriolae* UTF1 predicted genes, we found orthologs of virulence factors of pathogenic mycobacteria and human clinical *Nocardia* isolates involved in host cell invasion, modulation of phagocyte function and survival inside the macrophages. The virulence factor candidates provide an essential basis for understanding their pathogenic mechanisms at the molecular level by the fish nocardiosis research community in future studies. We also found many potential antibiotic resistance genes on the *N*. *seriolae* UTF1 chromosome. Comparative analysis with the four existing complete genomes, *N*. *farcinica* IFM 10152, *N*. *brasiliensis* HUJEG-1 and *N*. *cyriacigeorgica* GUH-2 and *N*. *nova* SH22a, revealed that 2,745 orthologous genes were present in all five *Nocardia* genomes (core genes) and 1,982 genes were unique to *N*. *seriolae* UTF1. In particular, the *N*. *seriolae* UTF1 genome contains a greater number of mobile elements and genes of unknown function that comprise the differences in structure and gene content from the other *Nocardia* genomes. In addition, a lot of the *N*. *seriolae* UTF1-specific genes were assigned to the ABC transport system. Because of limited resources in ocean environments, these *N*. *seriolae* UTF1 specific ABC transporters might facilitate adaptation strategies essential for marine environment survival. Thus, the availability of the complete *N*. *seriolae* UTF1 genome sequence will provide a valuable resource for comparative genomic studies of *N*. *seriolae* isolates, as well as provide new insights into the ecological and functional diversity of the genus *Nocardia*.

## Introduction

Members of the genus *Nocardia* are Gram-positive, non-motile and aerobic actinomycetes, belonging to the family Nocardiaceae. This genus contains more than 90 recognized species and are widely distributed in both aquatic and terrestrial habitats [1]. Many species of this genus are known as the causative agent of nocardiosis in humans and a variety of animals, which cause various clinical diseases and high mortality rates in some cases [2, 3]. In aquatic environments, four species of *Nocardia*, *N*. *asteroides*, *N*. *seriolae*, *N*. *salmonicida* and *N*. *crassostreae*, have also been found in diseased aquatic animals [4].

In Japan, members of the genus *Seriola*, including yellowtail (*S*. *quinqueradiata*), amberjack (*S*. *dumerili*), and kingfish (*S*. *lalandi*), are the most produced and economically important aquaculture fish species. Nocardiosis, caused by *N*. *seriolae* [5] (initially reported as *N*. *kampachi* [6]), is one of the most serious economic threats in the *Seriola* aquaculture. *N*. *seriolae* also infects other fish species including both marine and freshwater fishes and is found in other Asian countries [7]. To date, only two antibiotics, sulfamonomethoxine and sulfisozole sodium, are licensed for treatment of *N*. *seriolae* infections in Japan [8, 9]. Although these antibiotics are valuable for the control of nocardiosis, there are some concerns about the emergence of antibiotic-resistant strains and environmental impacts. Vaccination is thought to be another effective strategy for control of nocardiosis. However, the intracellular parasitic nature of *N*. *seriolae* makes development of vaccines for the disease difficult [10].

Complete genome sequences of pathogenic bacteria provide a powerful tool for understanding their biology, including mechanisms of bacterial pathogenicity and their drug-resistant properties, as well as for the development of new genetic and molecular approaches for disease control strategies [11]. So far, four *Nocardia* species have been fully sequenced, including three agents of human nocardiosis, *N*. *farcinica* IFM 10152 [12], *N*. *brasiliensis* HUJEG-1 [13] and *N*. *cyriacigeorgica* GUH-2 [14], and a rubber and gutta-percha-degrading strain, *N*. *nova* SH22a isolated from a root of *Couma macrocarpa* [15]. Although two draft sequences of *N*. *seriolae* isolates, ZJ0503 [16] and N-2927 [17] have been reported recently, these draft genome sequences consist of a large number of contigs, 319 contigs in 315 scaffolds for ZJ0503 [16] and 339 large contigs (>500bp) for N-2927 [17]. Therefore a complete genome sequence of *N*. *seriolae* is essential for a robust annotation, overall genome organization and comparative genomics of this species [18].

In this study, using Single Molecule, Real-Time (SMRT) DNA sequencing [19, 20], the complete genome nucleotide sequence of *N*. *seriolae* UTF1 isolated from a yellowtail that succumbed to nocardiosis in Japan was determined and annotated. We explored the virulence factors and antibiotic resistance gene candidates in the *N*. *seriolae* UTF1 genome. In addition, to investigate genomic diversity, the *N*. *seriolae* UTF1 genome sequence were compared with the four existing complete genomes of *Nocardia*. This is, to the best of our knowledge, the first report of the complete genome of the fish pathogenic *Nocardia* species. This genomic information will provide a reference genome data set of *N*. *seriolae* that could provide a basis for understanding the ecological and functional diversity of the genus *Nocardia* by comparative studies in future studies.

## Materials and methods

### Genome sequencing and assembly

*N*. *seriolae* UTF1 was originally isolated from a cultured yellowtail (*Seriola quinqueradiata*) in 2008 (Miyazaki Prefecture, Japan). This isolate was cultured in Brain Heart Infusion Broth (Difco, Sparks, MD, USA) at 25°C for 5 days under constant shaking with 150 rpm. After treatment with lysozyme, genomic DNA was extracted using the Maxwell Cell DNA Purification Kit (Promega, Madison, WI, USA). The nucleotide sequence of the *N*. *seriolae* UTF1 was determined by the Pacific Biosciences (PacBio) RS sequencing platform (Pacific Biosciences, Inc., CA, USA) at Tomy Digital Biology Co., Ltd (Tokyo, Japan). Briefly, genomic DNA (7 μg) was sheared using the g-TUBE (Covaris Inc., MA, USA) and a library was prepared using a DNA Template Prep Kit v2.0 (Pacific Biosciences) by the manufacturer’s instructions. The library was run in four Single Molecule, Real-Time (SMRT) cells on a PacBio RS sequencer (Pacific Biosciences) using P4C2 chemistry and a 120 minute data collection mode. The PacBio RS platform generated 227,796 sequence reads (mean read length: 4,756 bp, N50 read length: 7,364 bp) with 1,083,416,572 bp, providing a 133-fold sequencing coverage of the genome (Table 1). *De novo* assembly of sequence reads was performed using a SMRT Analysis v2.2.0 software package (Pacific Biosciences) and resulted in two contigs with lengths of 6,093,704 bp and 2,035,833 bp.

**Table 1:** Sequencing statistics of the *N*. *seriolae* UTF1 genome.

Number of Reads	227,796
N50 Read Length (bp)	7,364
Mean Read Length (bp)	4,756
Number of Bases	1,083,416,572
Average Reference Coverage	113.01
Number of contigs	2

For the genome sequence finishing, gaps between the two contigs were amplified by a long-range PCR method. The both gap-flanking sequences encoded ribosomal RNA (rRNA) operons, and therefore the PCR primer sets were designed for the outside of the region of rRNA operons. Using the gap-flanking PCR primer sets shown in S1 Table, long-range PCR was conducted using Phusion High-Fidelity PCR master mix with HF buffer (Thermo Fisher Scientific, Waltham, MA) following the manufacturer’s protocol. The long-range PCR amplification was performed using 50 ng of extracted total genomic DNA of *N*. *seriolae* UTF1 with an initial denaturation step of 30 s at 98°C and then a two-step PCR procedure (35 cycles of 98°C for 10 s and 72°C for 3 min), and 10 min of final extension at 72°C. The long-range PCR successfully amplified approximately 7–8 kb fragments correspond to the both gap regions and the two PCR products were purified with QIAquick PCR Purification Kit (QIAGEN, Hilden, Germany). The purified long-range PCR products (100 ng) were shotgun-sequenced using the Ion PGM platform (Thermo Fisher Scientific) and the sequence reads were assembled using V-GAP [21]. These two assembled sequences aligned to the two contigs, and consequently the complete nucleotide sequence of *N*. *seriolae* UTF1 comprising a circular chromosome was determined. The final sequence was submitted to DDBJ under accession number AP017900.

### Genome annotation

The complete genome sequence of *N*. *seriolae* UTF1was annotated using the Rapid Annotations using Subsystems Technology (RAST) server v2.0 with SEED data [22] and using BLASTP [23] against the NCBI RefSeq protein data [24] (*E* value threshold of 1*E*-5). Functional categories of the predicted genes of *N*. *seriolae* UTF1 and four other *Nocardia* spp. genes were assigned with the Clusters of Orthologous Groups of proteins (COGs) database [25] using COGsoft [26] and with the Kyoto Encyclopedia of Genes and Genomes (KEGG) pathway database using KEGG Orthology And Links Annotation (BlastKOALA) program [27]. Virulence factors of *N*. *seriolae* UTF1 were predicted by BLASTP against the Virulence Factor Database (VFDB) set A (a core dataset that covers genes associated with experimentally verified VFs) [28] and the virulence genes data set of *N*. *farcinica* IFM 10152 at the *Nocardia farcinica* Genome Project Page (http://nocardia.nih.go.jp/), using a cut off *E*-value of 1*E*−5. These BLAST results were then filtered using the criteria of query coverage per HSP (qcovhsp) greater than 80% and sequence similarity greater than 50%. The organization of the *mce* operon structures was identified based on the annotation result from the RAST server and visualized using Easyfig version 2.1 [29]. Genes for antibiotic resistance were estimated by Antibiotic Resistance Genes Database (ARDB) [30] with default parameters.

### Comparative genomics

To Identify and characterize the gap regions in the previous reported *N*. *seriolae* draft genome sequences, the contig sequences of *N*. *seriolae* ZJ0503 (GenBank: NZ_JNCT01000000, 319 contigs) and N-2927 (GenBank: NZ_BAWD02000000, 339 contigs) were aligned to the complete sequence of *N*. *seriolae* UTF1 genome using MUMmer version 3.22 [31]. The uncovered region sequences in the comparison with ZJ0503 and with N-2927 were subjected to BLASTX search against the NCBI RefSeq database (*E* value threshold of 1*E*−5).

Four available complete genome sequences of the genus *Nocardia*, *N*. *farcinica* IFM 10152 (GenBank: AP006618), *N*. *brasiliensis* HUJEG-1 (GenBank: CP003876), *N*. *cyriacigeorgica* GUH-2 (GenBank: FO082843) and *N*. *nova* SH22a (GenBank: CP006850), were used for the comparative genomic analysis with *N*. *seriolae* UTF1. For visualization of circular genome comparisons, the BLASTN-based ring image was generated by BLAST Ring Image Generator (BRIG) version 0.95 [32], with *N*. *seriolae* UTF1 as a reference. Dot plots of complete nucleotide sequences were generated by MUMmer version 3.22 and the mummerplot script and the Unix program gnuplot [31]. Average Nucleotide Identity (ANI) and Amino Acid Identity (AAI) were calculated using the ANI calculator and AAI calculator, respectively (default settings) [33]. The orthologous and species-specific genes were identified using OrthoMCL [34]. Comparisons of functional profiling of the *N*. *seriolae* UTF1 and four other *Nocardia* spp. complete genomes were carried out by the method by Verma et al. (2014) [35] with some modification. The top 50 SEED subsystems [36, 37] from the RAST analysis and the KEGG modules assigned by BlastKOALA were clustered hierarchically by the abundance of gene content for each categories among the *Nocardia* genomes using Cluster 3.0 software [38]. The results were visualized by Java Treeview version 1.1.6r4 [39].

## Results and discussion

### Assembly and general genomic features

The relatively large genome size (6–10 Mbp) with high GC contents (approximately 70%) of genus *Nocardia* [40] makes the completion of their genome sequences difficult. Because of the genomic complexity, draft assemblies of *N*. *seriolae* from an Illumina MiSeq platform [16] or a Roche 454-GS Junior System combined with the Illumina reads (NCBI Sequence Read Archive, DRX020602) [8, 17] consist of a large number of contigs. It should be noted that our initial draft sequence assembly of the *N*. *seriolae* UTF1 genomes using a 454 GS-FLX+ System resulted in a total of 134 scaffolds comprising 365 contigs (unpublished data). The PacBio RS platform, a third-generation sequencing technology and based on single-molecule real-time (SMRT) sequencing, can achieve unbiased GC coverage with extremely long reads [19, 20]. This platform has been employed successfully for sequencing in complex bacterial genomes such as those with extremely high GC content genomes [41] and with multiple chromosomes containing more repetitive sequences [42]. In this study, we determined the complete genome nucleotide sequence of *N*. *seriolae* UTF1 using the PacBio RS.

**Fig 1:**
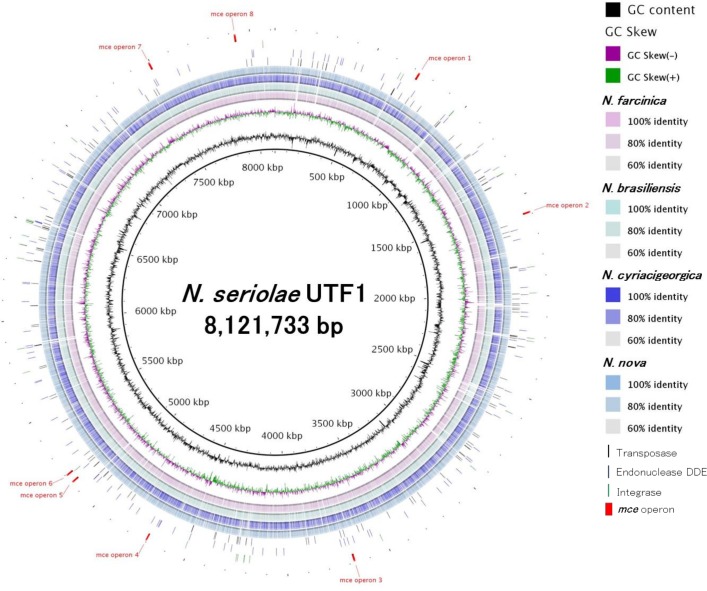
Comparative genomic map of *N*. *seriolae* UTF1 genome and the other four *Nocardia* complete genomes. The BLASTN-based ring image was generated by BLAST Ring Image Generator (BRIG) version 0.95 [32]. The innermost two rings show GC content (black) and GC skew (purple/green). The remaining rings (rings 3–6) represent a BLASTN comparison with complete genome of *N*. *farcinica* IFM 10152 (magenta), *N*. *brasiliensis* HUJEG-1(cyan), *N*. *cyriacigeorgica* GUH-2 (blue) and *N*. *nova* SH22a (pale blue). Bars indicate the position of mobile-element related genes in the *N*. *seriolae* UTF1 genome such as transposases (black), endonuclease DDE (blue) and integrase (green). The outermost red boxes highlight the 8 *mce* operons found in the *N*. *seriolae* UTF1 genome.

Our *de novo* assembly with a 133-fold genome coverage of PacBio RS long-reads (mean read length: 4,756 bp, N50 read length: 7,364 bp) produced two large contigs with lengths of 6,093,704 bp and 2,035,833 bp (Table 1). The flanking sequences at both sides of the two assembled contigs encoded ribosomal RNA genes. Since the length of multiple copies of rRNA operons (16S-23S-5S rRNA) are approximately 5 kb, the current sequence reads with an average of 4,756 bp may have not been able to fully cover these rRNA operon regions. It should be noted that the newest PacBio RS II sequencer generates an average read length of 10–15 kb [43], and therefore the new sequencer is assumed to be able to assemble around these rRNA operon regions. After closing the gaps with a long-range PCR method, the complete nucleotide sequence of *N*. *seriolae* UTF1 comprising a circular chromosome of 8,121,733 bp with a G+C content of 68.1% was determined (Fig 1 and Table 2). The complete genome of *N*. *seriolae* UTF1 contains 7,697 predicted coding DNA sequences (CDSs) with an average length of 909 bp, 4 rRNA operons, and 62 transfer RNA (tRNA) sequences (Table 2). The genome sizes vary among the fully sequenced *Nocardia* genomes that range from 6,021,225 bp for *N*. *farcinica* IFM 10152 to 9,436,348 bp for *N*. *brasiliensis* HUJEG-1, while the numbers of CDSs also vary among them that range from 5,491 for *N*. *farcinica* IFM 10152 to 8,414 for *N*. *brasiliensis* HUJEG-1 (Table 2). On the other hand, the *N*. *seriolae* UTF1 genome has four rRNA operons and 62 tRNAs, while three rRNA operons and 49–53 tRNAs have been found in the other four fully sequenced *Nocardia* genomes (Table 2).

**Table 2:** Comparison of genomic features of *N*. *seriolae* UTF1 and the other four *Nacardia* spp. complete genomes.

Species	host	Accesion number	Size (bp)	GC%	CDS	rRNA	tRNA	Reference
*N*. *seriolae* UTF1	*Seriola quinqueradiata*	AP017900	8,121,733	68.1	7,697	4	62	This study
*N*. *farcinica* IFM 10152	*Homo sapiens*	AP006618	6,021,225	70.8	5,674	3	53	[12]
*N*. *brasiliensis* HUJEG-1	*Homo sapiens*	CP003876	9,436,348	68.0	8,414	3	51	[13]
*N*. *cyriacigeorgica* GUH-2	*Homo sapiens*	FO082843	6,194,645	68.4	5,491	3	49	[14]
*N*. *nova* SH22a	A root of *Couma macrocarpa*	CP006850	8,348,532	67.8	7,583	3	49	[15]

The genome sequences of *N*. *seriolae* UTF1 and the previously reported *N*. *seriolae* isolates were quite similar. The *N*. *seriolae* UTF1 genome showed 99.99 and 99.95% ANI with ZJ0503 [16] and N-2927 [17], respectively. It should be noted that the ANI between N-2927 and U-1 [8], the most recently reported draft genome of *N*. *seriolae* isolate, was 100%. Therefore, we did not use the U-1 genome for further comparisons and analysis. To clarify the uncovered regions (gaps) in the previously reported *N*. *seriolae* genome sequences, the contig sequences of *N*. *seriolae* ZJ0503 [16] (319 contigs) and N-2927 [17] (339 contigs) were aligned to the complete sequence of *N*. *seriolae* UTF1 genome. As a result, 300 uncovered regions (average length: 1,373 bp) were detected in the draft genome of ZJ0503, while 297 uncovered regions (average length: 1,502 bp) were detected in the draft genome of N-2927. These gap sequences were subjected to BLASTX searches against the NCBI RefSeq database (*E* value threshold of 1*E*−5) and as a result, 294 (98.0%) for ZJ0503 and 290 (97.6%) for N-2927 had significant BLAST hits. The BLAST results revealed that most assigned genes (76.9% for ZJ0503 and 73.1% for N-2927) were mobile element-related, such as for transposase, endonuclease DDE and integrase (S1 Fig). The comparative genomic map of *N*. *seriolae* UTF1 with ZJ0503 and N-2927 also showed that these mobile element-related genes are interspersed across the *N*. *seriolae* UTF1 genome and coincides with the gap regions of ZJ0503 and N-2927 (S2 Fig). Since the nucleotide length of these genes was more than 1kb, these repeat sequences have a significant influence during *de novo* assembly when relatively short reads (several hundred bp) from MiSeq and 454-GS Junior are used [8, 16, 17]. In the present study, the PacBio long-reads (mean read length: 4,756 bp, N50 read length: 7,364 bp) could cover over these repeated sequences and achieve completion of the *N*. *seriolae* UTF1 genome.

### Overview of *N*. *seriolae* UTF1 virulence factors

Like causal agents of human nocardiosis, *N*. *seriolae* is considered to be an intracellular pathogen that invades and grows within host cells, even including phagocytes [44, 45]. The intracellular nature of this bacteria makes it difficult to control disease. As a first step toward understanding the virulence factors and pathogenic properties of *N*. *seriolae* UTF1, we conducted BLASTP searches of *N*. *seriolae* UTF1 CDSs against the Virulence Factor Database (VFDB) [28] and a well-annotated virulence genes data set of *N*. *farcinica* (http://nocardia.nih.go.jp/) (*E*-value < 1*E*-5, sequence length overlap > 80% and sequence similarity > 50%). The VFDB search identified 173 CDSs as candidate virulence factors (S2 Table). In addition, almost all of *N*. *farcinica* putative virulence genes were found in the *N*. *seriolae* UTF1 genome (Table 3).

**Table 3:** Virulence factor candidates in the *N*. *seriolae* UTF1 by comparison with the virulence genes data set of *N*. *farcinica*.

Category	*N*. *seriorae* UTF1 ORF ID	*N*. *farcinica* gene ID*	Gene*	Description*	%Identity	%Similarity	*E* value
Cell wall proteins	ORF-145	nfa1810	*fbpA*	mycolyltransferase	70.9	79.9	5.00E-159
	ORF-7590	nfa1820	*fbpB*	mycolyltransferase	56.4	75.9	7.00E-67
	ORF-150	nfa1830	*fbpC*	mycolyltransferase	57.7	73.0	2.00E-37
Metal importers	ORF-5098	nfa37790	*ideR*	transcriptional regulator	66.0	79.4	4.00E-84
	ORF-950	nfa7630	*nbtA*	thioesterase	85.7	90.2	0
	ORF-951	nfa7640	*nbtB*	polyketide synthase	86.4	90.0	5.00E-60
	ORF-952	nfa7650	*nbtC*	polyketide synthase	87.3	93.0	1.00E-143
	ORF-953	nfa7660	*nbtD*	non-ribosomal peptide synthetase	88.5	93.8	0
	ORF-954	nfa7670	*nbtE*	non-ribosomal peptide synthetase	91.6	96.2	4.00E-161
	ORF-750	nfa7680	*nbtF*	non-ribosomal peptide synthetase	93.0	97.1	2.00E-108
	ORF-948	nfa7610	*nbtG*	lysine-N-oxygenase	84.7	93.1	0
	ORF-747	nfa6190	*nbtS*	salicylate synthase	81.4	88.5	4.00E-126
	ORF-749	nfa6200	*nbtT*	salicylate-AMP ligase	82.5	91.3	4.00E-126
Oxidative and nitrosative stresses	ORF-5118	nfa37890	*ahpC*	alkylhydroperoxide reductase	68.0	79.0	5.00E-149
	ORF-5119	nfa37900	*ahpD*	alkylhydroperoxidase	68.8	79.3	0
	ORF-325	nfa55390	*katC*	catalase	79.0	87.8	0
	ORF-4212	nfa29500	*katG*	catalase-peroxidase	63.8	77.8	1.00E-169
	ORF-6237	nfa45490	*narG*	nitrate reductase alpha subunit	71.3	84.8	0
	ORF-6238	nfa45500	*narH*	nitrate reductase beta subunit	72.4	83.3	1.00E-78
	ORF-6240	nfa45520	*narI*	nitrate reductase gamma subunit	75.3	84.6	0
	ORF-6239	nfa45510	*narJ*	nitrate reductase delta subunit	74.1	83.8	1.00E-157
	ORF-6265	nfa45610	*nirB*	nitrite reductase (NAD(P)H) subunit	76.9	86.9	0
	ORF-6264	nfa45600	*nirD*	nitrite reductase (NAD(P)H) subunit	75.6	84.0	1.00E-173
	ORF-5117	nfa37880	*oxyR*	hydrogen peroxide sensing transcriptional regulator	66.9	78.6	2.00E-165
	ORF-418	nfa52980	*sodC*	superoxide dismutase	78.2	86.4	1.00E-140
	ORF-68	nfa1210	*sodF*	superoxide dismutase	40.5	57.5	3.00E-112
Penetration into mammalian cells	ORF-4357	nfa34810	*inv*	invasin	65.6	80.4	2.00E-119
Phagosome arresting	ORF-5937	nfa13510	*ndk*	nucleoside diphosphate kinase	44.8	57.6	2.00E-45
	ORF-5498	nfa16310	*ptpA*	protein-tyrosine phosphatase	48.7	61.4	2.00E-88
	ORF-2644	nfa18680	*ptpB*	protein-tyrosine phosphatase	60.2	70.7	0
other	ORF-2873	nfa19960	*tlyA*	putative cytotoxin/hemolysin	77.4	83.2	6.00E-135

* according to the Nocardia farcinica Genome Project Page (http://nocardia.nih.go.jp/).

**Fig 2:**
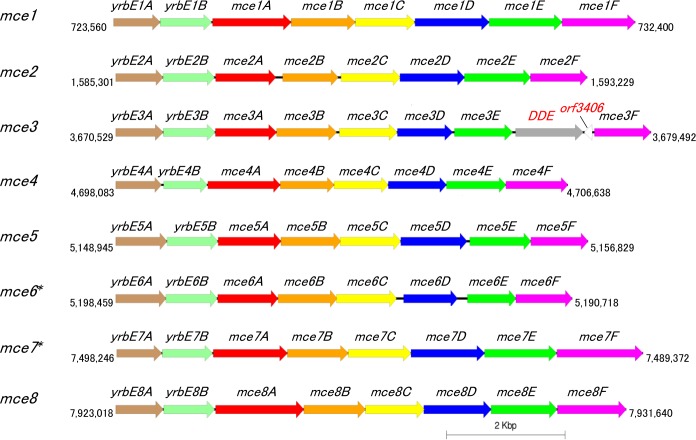
The organization of 8 *mce* operons in *N*. *seriolae* UTF1 genome. The figure was generated using Easyfig 2.1 [29]. Arrows represent *yrbE* genes, *mce* genes and two additional genes, DDE (endonuclease DDE) and *orf3406* (hypothetical protein) in *mce3*. Values indicate the number of base positions. Asterisks (mce6* and mce7*) indicate reverse complement orientation.

Mammalian cell entry (Mce)-family proteins, virulence factors of *Mycobacterium tuberculosis* (the class Actinobacteria), have the ability to enter into mammalian cells and survive inside the macrophage [46]. The genome of *M*. *tuberculosis* contains four *mce* operons which comprise eight genes per operon in identical manner (two *yrbE* genes, A and B; six *mce* genes, A, B, C, D, E and F) [47, 48]. Mce proteins are found in diverse Actinobacteria including *Nocardia* spp. [49]. Six copies of *mce* operons have been found in the three human clinical isolates (*N*. *farcinica* IFM 10152 [12], *N*. *brasiliensis* HUJEG-1 [13] and *N*. *cyriacigeorgica* GUH-2 [14]), whereas 14 *mce* operons have been found in *N*. *nova* SH22, which was isolated from a plant root and has the ability of rubber and gutta-percha degradation [15]. Recently, Carrillo-González et al. (2016) demonstrated the importance of *mce* proteins for *Nocardia* pathogenesis from whole-genome comparison of an attenuated *N*. *brasiliensis* HUJEG-1 and the parental strain [50]. In the *N*. *seriolae* UTF1 genome, we found eight complete *mce* loci with nucleotide length of 6,814 bp to 8,964 bp (Figs 1 and 2). It should be noted that the locus *mce3* has two extra genes (endonuclease *DDE* and *orf3406*) between *mce3E and mce3F* (Fig 2). However, the influence of these two extra genes to the function of the *mce3* operon is unclear. Amino acid sequence similarities of *N*. *seriolae* UTF1 Mce1 proteins with the other four *Nocardia* species, and two Actinobacteria, *Rhodococcus equi* and *M*. *tuberculosis* are shown in S3 Table. The Mce1C protein is highly conserved among Mce1 proteins (87.8–90.7% similarities with *Nocardia* spp., 82.0% similarity with *R*. *equi* and 63.8% similarity with *M*. *tuberculosis*), while the Mce1E protein exhibit a relatively lower sequence homology (75.4–80.0% similarities with *Nocardia* spp., 75.3% similarity with *R*. *equi* and 63.3% similarity with *M*. *tuberculosis*). Invasin also plays a role in attachment and penetration into host cells by several bacterial species [51, 52], *Nocardia* species also possess an invasin gene [12, 13] and the *N*. *seriolae* UTF1 ORF-4357 is very similar to *N*. *farcinica* IFM 10152 invasin (80.4% similarity) (Table 3). Since *Nocardia* species are facultative intracellular pathogens, they most likely use this protein for entry into host cells. Further studies to determine the function of *Nocardia* invasin involved in the host cell entry are required.

*Nocardia* species can modulate phagocyte function and can grow in macrophages. It has been reported that catalase and superoxide dismutase (SOD) impair the ability of the oxidative killing mechanisms of phagocytes [53]. In the *N*. *seriolae* UTF1 genome, two catalases (*katC*, ORF-325; *katG*, ORF-4212) and two *sod* (*sodC*, ORF-418; *sodF*, ORF-68) genes are present (Table 3). We also found the *N*. *seriolae* UTF1 CDSs that are homologous to the *N*. *farcinica* IFM 10152 nitrate reductase genes, *narG* (84.8%), *narH* (83.3%), *narI* (84.6%), *narJ* (83.8%), *nirB* (86.9%) and *nirD* (84.0%) (Table 3), which may contribute to survival under low-oxygen conditions in stimulated macrophages [54, 55]. Recently, it has been reported that the attenuated *N*. *brasiliensis* HUJEG-1 lost a catalase, SOD and several nitrate reductase genes, suggesting that these genes may be associated to *Nocardia* spp. pathogenesis [50]. In addition to the above oxidative and nitrosative stress-related genes, alkylhydroperoxidases, AhpC and AhpD, have a crucial role for antioxidant defense in mycobacterial species [56, 57], particularly when the KatG catalase-peroxidase activity is depressed [58]. In the AhpC/AhpD antioxidant defense system, OxyR acts as a positive regulator for their expression [59]. The *N*. *seriolae* UTF1 genome contains these genes similar to *N*. *farcinica* IFM 10152 with a protein homology of *ahpC* (79.0%), *ahpD* (79.3%) and *oxyR* (78.6%) (Table 3).

A phagosome, a cellular compartment, is essential for intracellular killing and digesting of pathogenic microorganisms [60]. Nucleoside diphosphate kinase (Ndk) and protein tyrosine phosphatase A (PtpA) arrest macrophage phagosomal maturation for the intracellular survival and persistence of pathogenic mycobacteria [61]. The *N*. *seriolae* UTF1 ORF-5937 and ORF-5498 are homologous to *N*. *farcinica* IFM 10152 *ndk* (57.6%) and *ptpA* (61.4%), respectively (Table 3). Since iron is in very low concentration and in an insoluble state within macrophages, efficient iron-acquisition systems are required for pathogenic bacteria to survive [61]. Putative *N*. *seriolae* UTF1 metal importer related genes (*ideR* and *nbtA–G*, *S*, *T*) were identified (Table 3), and may contribute to the abilities of *N*. *seriolae* to survive in fish tissues including within macrophages.

Other virulence factor candidates were also found in the *N*. *seriolae* UTF1 genome. Antigen 85 (Ag85) complex of *M*. *tuberculosis* is a family of fibronectin binding proteins (Fbp) that plays an essential role in the pathogenesis of tuberculosis [62]. The Ag85 complex consists of three proteins (Ag85A, Ag85B and Ag85C: encoded by the genes *fbpA*, *fbpB* and *fbpC*) that possess mycolyltransferase activity involved in the final stages of mycobacterial cell wall assembly [63]. The *N*. *seriolae* UTF1 genome was found to have at least seven putative *fbp* genes: three *fbpA* (ORF-144, ORF-145 and ORF-147), two *fbpB* (ORF-146 and ORF-7590) and two *fbpC* (ORF-148 and ORF-150). Among these seven *fbp* gene candidates, protein sequences of ORF-145 (*fbpA*), ORF-7590 (*fbpB*) and ORF-150 (*fbpC*) were most similar to *N*. *farcinica* IFM 10152 *fbpA* (79.9%), *fbpB* (75.9%) and *fbpC* (73.0%) (Table 3). TlyA proteins of the pathogenic bacteria has a virulent hemolytic ability [64, 65], and the *N*. *seriolae* UTF1 ORF-2873 encodes a TlyA, with a homology of 83.2% to the *N*. *farcinica* IFM 10152 (Table 3). In contrast to the PtpA (mentioned above), PtpB is considered to be nonessential for the phagosome arresting function of *M*. *tuberculosis* [61]. On the other hand, Zhoua et al. (2010) reported that *M*. *tuberculosis* PtpB depresses the innate immune responses by inhibiting the signaling pathway involved in interleukin-6 (IL-6) production and promoting host cell survival by activating the Akt pathway for their survival in macrophages [66]. The *N*. *seriolae* UTF1 genome contains a *ptpB* gene (ORF-2644), with a similar protein in *N*. *farcinica* IFM 10152 (70.7%) (Table 3).

Overall, the whole-genome analysis of *N*. *seriolae* UTF1 reveals that the genome contains known virulence genes of mycobacteria and human clinical *Nocardia* isolates for host cell invasion, modulation of phagocyte function and surviving inside the macrophages. Therefore, the presence of these virulence genes in the *N*. *seriolae* UTF1 may explain their ability to survive intracellularly and within macrophages. The virulence gene set of *N*. *seriolae* UTF1 we present provides the material basis for further study of their pathogenic mechanisms at the molecular level. In addition, the complete genome sequence of *N*. *seriolae* UTF1 can be utilized for comparing the genomes between pathogenic and non (less)-pathogenic isolates to more fully resolve the genes responsible for *N*. *seriolae* pathogenesis in future studies.

### Potential antibiotic resistance genes of *N*. *seriolae* UTF1

In general, *Nocardia* spp. are naturally resistant to many antibiotics and most β-lactams [7, 67–69]. The *N*. *seriolae* UTF1 encodes at least 14 β-lactamases, while the number of β-lactamase genes is one for *N*. *farcinica* IFM 10152 [12], 29 for *N*. *brasiliensis* HUJEG-1 [13] and 12 for *N*. *cyriacigeorgica* GUH-2 [14]. In addition, as found in the draft genome of *N*. *seriolae* genomes [8, 16, 17], the *N*. *seriolae* UTF1 genome has one vancomycin and two fluoroquinolones resistant gene candidates according to the RAST annotation. To explore more antibiotic resistant genes in the *N*. *seriolae* UTF1 chromosome, all their CDSs were compared with the Antibiotic Resistance Genes Database (ARDB) [30]. This analysis identified 20 CDSs as candidate antibiotic resistant genes that were classified into 16 antibiotic resistance gene types (Table 4). The presence of these antibiotic resistance genes of the *N*. *seriolae* UTF1 may, in part, explain the difficulty in treating diseases caused by *N*. *seriolae*.

**Table 4:** Potential antibiotic resistance genes of *N*. *seriolae* UTF1 according to the Antibiotic Resistance Genes Database (ARDB).

Resistance gene type	Antibiotic resistance	Description	UTF1 ORF ID	Best Hit Accession	*E*-Value	Score
*aac(6’)-Ic*	Amikacin, dibekacin, isepamicin, netilmicin, sisomicin, tobramycin	Aminoglycoside N-acetyltransferase, which modifies aminoglycosides by acetylation.	ORF-1348	AAA26549	6.00E-32	130
*bacA*	Bacitracin	Undecaprenyl pyrophosphate phosphatase, which consists in the sequestration of Undecaprenyl pyrophosphate.	ORF-4188	CAL13705	8.00E-23	101
*bl2a_iii2*	Penicillin	Class A beta-lactamase. This enzyme breaks the beta-lactam antibiotic ring open and deactivates the molecule's antibacterial properites.	ORF-6138	YP_089932	8.00E-65	241
*carA*	Lincosamide, macrolide, streptogramin b	ABC transporter system, Macrolide-Lincosamide-Streptogramin B efflux pump.	ORF-4637	AAC32027	2.00E-10	58
*catbB1*	Chloramphenicol	Group B chloramphenicol acetyltransferase, which can inactivate chloramphenicol. Also referred to as xenobiotic acetyltransferase.	ORF-1707	AAK88712	3.00E-06	45
*dfrA26*	Trimethoprim	Group A drug-insensitive dihydrofolate reductase, which can not be inhibited by trimethoprim.	ORF-6939	CAL48457	8.00E-29	120
*macB*	Macrolide	Resistance-nodulation-cell division transporter system. Multidrug resistance efflux pump. Macrolide-specific efflux system.	ORF-1987	YP_001453760	9.00E-54	204
			ORF-4813	YP_001571041	1.00E-46	180
			ORF-6113	YP_001334578	5.00E-47	181
			ORF-6376	YP_001453760	2.00E-49	189
*mfpA*	Fluoroquinolone	Pentapeptide repeat family, which protects DNA gyrase from the inhibition of quinolones.	ORF-3773	ABL05132	6.00E-08	52
*otr*(B)	Tetracycline	Major facilitator superfamily transporter, tetracycline efflux pump.	ORF-1750	AAD04032	1.00E-105	375
*srmB*	Lincosamide, macrolide, streptogramin b	ABC transporter system, Macrolide-Lincosamide-Streptogramin B efflux pump.	ORF-4948	CAA45050	9.00E-68	250
*tcmA*	Tetracenomycin c	Major facilitator superfamily transporter. Resistance to tetracenomycin C by an active tetracenomycin C efflux system which is probably energized by transmembrane electrochemical gradients.	ORF-4285	AAA67509	3.00E-05	40
*tcr3*	Tetracycline	Major facilitator superfamily transporter, tetracycline efflux pump.	ORF-6293	BAA07390	8.00E-99	355
*vanRB*	Vancomycin	VanB type vancomycin resistance operon genes, which can synthesize peptidoglycan with modified C-terminal D-Ala-D-Ala to D-alanine—D-lactate.	ORF-5538	ABB53368	1.00E-13	70
			ORF-7016	ABB53368	3.00E-16	79
*vanRC*	Vancomycin	VanC type vancomycin resistance operon genes, which can synthesize peptidoglycan with modified C-terminal D-Ala-D-Ala to D-alanine—D-serine.	ORF-2855	AAY67971	3.00E-31	129
*vatB*	Streptogramin a	Virginiamycin A acetyltransferase, which can inactivate the target drug.	ORF-4237	YP_001038094	9.00E-10	57
*vatC*	Streptogramin a	Virginiamycin A acetyltransferase, which can inactivate the target drug.	ORF-3078	AAG21695	6.00E-49	187

It has been reported that *N*. *seriolae* isolates could be divided into two phenotypic groups using α-glucosidase (α-glu) activity (α-glu-positive or -negative) [7, 68]. These two groups showed different oxytetracycline (OTC: a tetracyclinic antibiotics)/erythromycin (Em: a macrolide antibiotic) susceptibility profiles. Most of the α-glu-positive isolates were OTC-resistant and Em-sensitive, while most of α-glu-negative isolates were OTC-sensitive and Em-resistant [69,70]. *N*. *seriolae* UTF1 was α-glu-positive isolate which exhibited resistance to OTC and sensitivity to Em. Ismail et al. 2011 [69] found that OTC-resistant strains of *N*. *seriolae* possess *tet(K)* and/or *tet(L)* gene(s), while the Em-resistant strains possessed *mef(A)* and *msr(D)* genes. The *tet(K)* and *tet(L)* genes are generally found on small transmissible plasmids [71,72], while *mef(A)* and *msr(D)* genes are encoded in chromosomes of Gram-positive bacteria and associated with conjugative transposons [73]. Despite obtaining a 1.1 Gbp (133-fold genome coverage) of PacBio RS long-reads in this study and a 247 Mbp (30-fold genome coverage) of 454 reads (unpublished data), we could not find any plasmids or *tet(K)* and *tet(L)* gene sequences. The absence of these genes might have been caused by plasmid elimination during culture propagation. On another front, the *N*. *seriolae* UTF1 chromosome was found to have two tetracycline resistance gene candidates, *otr*(B) (ORF-1750) and *tcr3* (ORF-6293) (Table 4), both found in *Streptomyces* spp. (Actinobacteria) [71], suggesting that these two genes might enhance some degree of resistance to OTC in *N*. *seriolae*.

As expected, *mef(A)* and *msr(D)* genes were not found in the *N*. *seriolae* UTF1 chromosome. However, the results from ARDB includes three candidates for macrolide efflux pump genes: *carA* (ORF-4637), *macB* (ORF-1987, ORF-4813, ORF-6113 and ORF-6376) and *srmB* (ORF-4948) (Table 4). Since *N*. *seriolae* UTF1 is an Em-sensitive isolate, these three candidates are not likely to be involved in Em-resistance of *N*. *seriolae* UTF1. In particular, MacB requires TolC and MacA for its function in *E*. *coli* [74,75], but the *N*. *seriolae* UTF1 genome lacks both genes. Thus, further information on the antibiotic profile of more *N*. *seriolae* isolates, as well as their genomic sequences are needed for an accurate view of their antibiotic resistance genes, and the complete genome sequence of the *N*. *seriolae* UTF1 can be used as a reference for these surveys in future studies.

### Genome comparison with fully sequenced genomes of the genus *Nocardia*

Four available complete genome sequences of the genus *Nocardia*; *N*. *farcinica* IFM 10152, *N*. *brasiliensis* HUJEG-1, *N*. *cyriacigeorgica* GUH-2 and *N*. *nova* SH22a, were used for comparison of the genomic structure with the *N*. *seriolae* UTF1. From the comparative genomic map (Fig 1), we found that there were no large-scale variations among the genomes, but a considerable number of non-homologous regions are scattered around the *N*. *seriolae* UTF1 genome. Most of these non-homologous regions were linked to mobile element-related genes (transposase, endonuclease DDE and integrase) (Fig 1), suggesting they serve as plastic and variable regions for the *N*. *seriolae* UTF1 genome.

**Fig 3:**
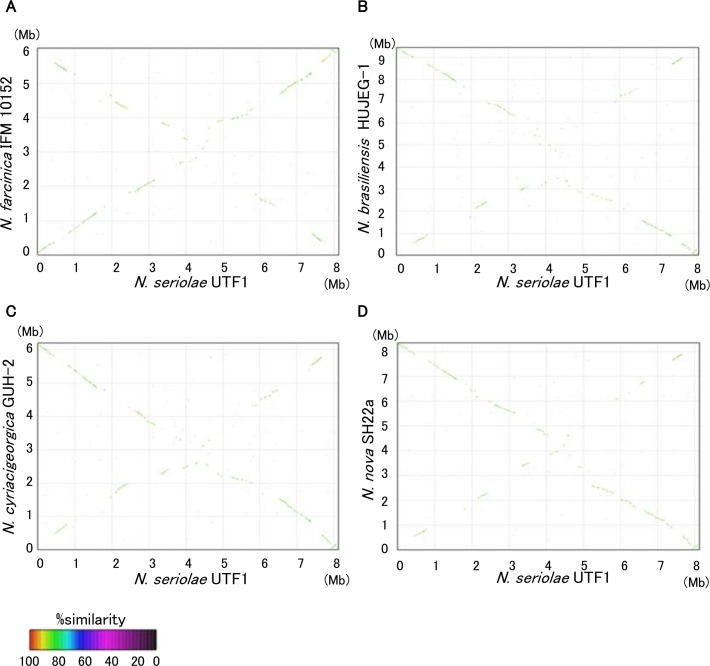
**Dot plot comparisons of *N*. *seriolae* UTF1 genome against *N*. *farcinica* IFM 10152 (A), *N*. *brasiliensis* HUJEG-1 (B), *N*. *cyriacigeorgica* GUH-2 (C) and *N*. *nova* SH22a (D).** Nucleotide-based alignments were performed with MUMmer version 3.22 and dot plots were generated by the mummerplot script and the Unix program gnuplot [31].

The whole-genome alignments of *N*. *seriolae* UTF1 with the other four *Nocardia* displayed an X-shaped distribution across the origin of replication (Fig 3), which is explained by the fork replication theory [76]. By contrast, the alignments of the three agents of human nocardiosis; *N*. *farcinica* IFM 10152, *N*. *brasiliensis* HUJEG-1 and *N*. *cyriacigeorgica* GUH-2, showed only a few visible symmetric inversions and a diagonal line with a slope of approximately 1 (S3 Fig). On the other hand, the alignments of *N*. *nova* SH22a and the three human clinical isolates were arranged in an X-shaped distribution as in the case of *N*. *seriolae* UTF1. Thus, the degree of symmetrical inversions indicates that the genetic distance among the three human clinical isolates are close to each other, while *N*. *seriolae* UTF1 and *N*. *nova* SH22a are genetically distant from these species.

The ANI and AAI based pairwise comparisons between each genome are shown in Table 5. Typically, the ANI values between genomes of the same bacterial species show above 95%, while the values below 75% are too divergent to be compared based on this measurement [33]. For the latter case, AAI provides a much more robust resolution. AAI cut-offs for genus and species boundary have been estimated to be 55–60% and 85–90%, respectively [33]. The ANI and AAI between *N*. *seriolae* UTF1 and the other four *Nocardia* genomes ranged between 79.21–79.88% and 68.96–69.62%, respectively. Like the results of whole-genome alignments, the three human clinical isolates showed higher ANI and AAI values (81.19–81.61% ANI and 73.61–74.99% AAI) than the comparisons with *N*. *nova* SH22a (79.43–79.89% ANI and AAI (68.06–69.82%) and with *N*. *seriolae* UTF1 (described above). On the other hand, the ANI and AAI between *N*. *seriolae* UTF1 and *N*. *nova* SH22a had relatively low values, that displayed 79.63% ANI and 69.17% AAI, respectively. Overall, the ANI and AAI values indicate that the three human clinical isolates are a genetically similar group and *N*. *seriolae* UTF1, an agent of fish nocardiosis, is genetically distant from these species as well as *N*. *nova* SH22, an environmental isolate.

**Table 5:** Average nucleotide identity (ANI, upper grids) and average amino acid identity (AAI, lower grids) values (in percent) calculated between *Nocardia* genomes.

	*N*. *seriolae* UTF1	*N*. *farcinica* IFM 10152	*N*. *brasiliensis* HUJEG-1	*N*. *cyriacigeorgica* GUH-2	*N*. *nova* SH22a
***N*. *seriolae* UTF1**	-	79.88	79.21	79.88	79.63
***N*. *farcinica* IFM 10152**	69.17	-	81.36	81.61	79.77
***N*. *brasiliensis* HUJEG-1**	68.96	73.61	-	81.19	79.43
***N*. *cyriacigeorgica* GUH-2**	69.62	74.99	73.68	-	79.89
***N*. *nova* SH22a**	69.17	69.21	68.06	69.82	-

**Fig 4:**
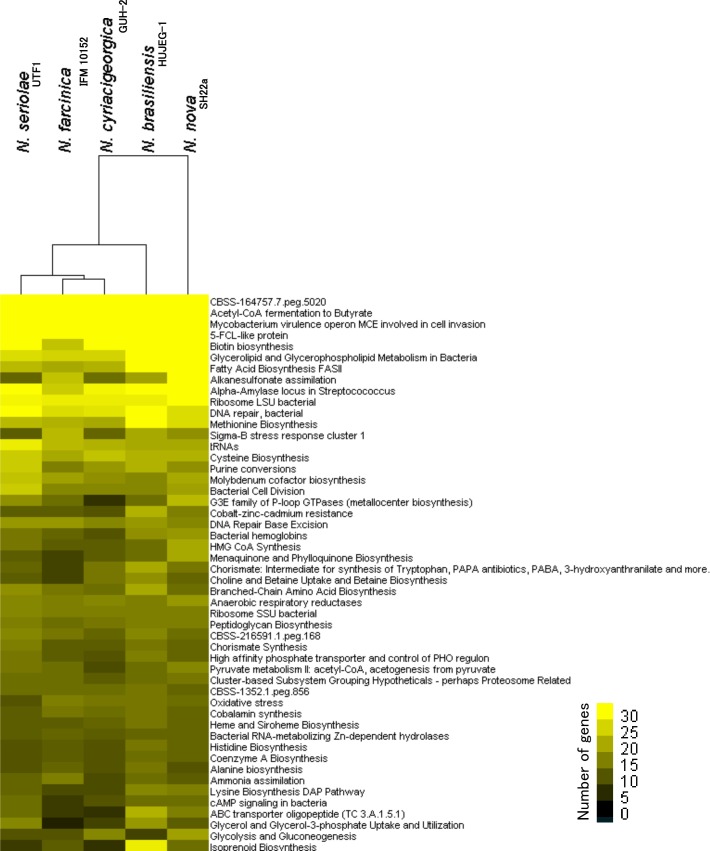
Functional profiling of the five *Nocardia* complete genomes. Heat map shows the abundance of the top 50 subsystems [36, 37] enriched in the five *Nocardia* genomes. The color scale indicates the abundance of gene content for each category.

We also focused on similarity of the functional profiling among the five complete *Nocardia* genomes. Fig 4 shows a dendrogram constructed based on the top 50 subsystems from the RAST analysis. Interestingly, although a distant genetic relationship was observed between *N*. *seriolae* UTF1 and the three agents of human nocardiosis through whole genome comparisons, the analysis revealed that the functional repertoire of *N*. *seriolae* UTF1 is closer to the three agents of human nocardiosis than *N*. *nova* SH22a (Fig 4). Similar results were obtained with the dataset of overall subsystems (S4 Fig) and the KEGG modules assigned by BlastKOALA (S5 Fig). These findings suggest that the closer functional relationship between the agents of the human and fish nocardiosis may be associated with their adaptations to infected animal hosts and pathogenic properties.

In summary, when the five complete genomes of *Nocardia* are compared, we found that *N*. *seriolae* UTF1 is genetically distant from the three agents of human nocardiosis, but is similar to the functional repertoire of them. Further research studies are required to more fully resolve the phylogenetic relationship of *Nocardia* spp. [40] and the differentially enriched pathways according to their habitat and lifestyle, when a greater number of completely sequenced and thoroughly annotated clinical and environmental *Nocardia* isolates become available.

### Finding functional features in *N*. *seriolae* UTF1 genome

**Fig 5:**
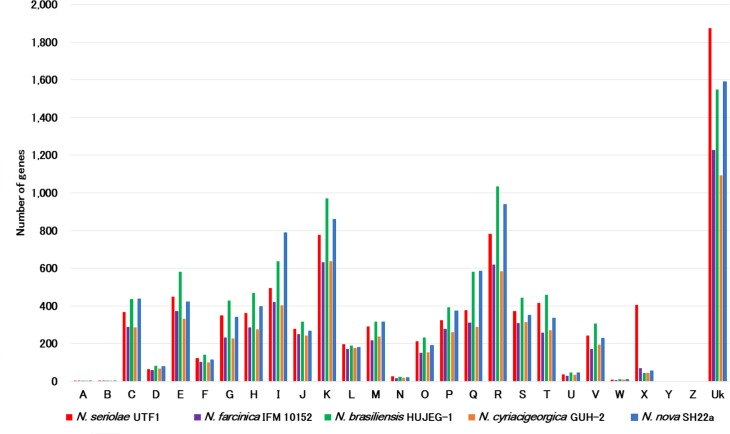
Comparison of COG distribution of *N*. *seriolae* UTF1 and the other four *Nocardia* genomes. COG definitions are described as follows: A, RNA processing and modification; B, Chromatin structure and dynamics; C, Energy production and conversion; D, Cell cycle control, cell division, chromosome partitioning; E, Amino acid transport and metabolism; F, Nucleotide transport and metabolism; G, Carbohydrate transport and metabolism; H, Coenzyme transport and metabolism; I, Lipid transport and metabolism; J, Translation, ribosomal structure and biogenesis; K, Transcription; L, Replication, recombination and repair; M, Cell wall/membrane/envelope biogenesis; N, Cell motility; O, Posttranslational modification, protein turnover, chaperones; P, Inorganic ion transport and metabolism; Q, Secondary metabolites biosynthesis, transport and catabolism; R, General function prediction only; S, Function unknown; T, Signal transduction mechanisms; U, Intracellular trafficking, secretion, and vesicular transport; V, Defense mechanisms; W, Extracellular structures; X, Mobilome: prophages, transposons; Y, Nuclear structure; Z, Cytoskeleton. ‘Uk’ indicate unknown (unassigned genes). The number of ORFs with each COG category are listed in S4 Table.

The complete sequence of the *N*. *seriolae* UTF1 genome has been determined for the first time as a marine *Nocardia* species, and therefore it is interesting to investigate the characteristic features of its genome. According to the COG classifications, the distribution of COG categories is mostly similar among *Nocardia* genomes (Fig 5). In the *N*. *seriolae* UTF1, the number of genes for the category ‘Mobilome: prophages, transposons (X)’ and ‘Unknown’ (not assignable to COG categories) is higher than that in the other four *Nocardia* genomes (Fig 5). Notably, genes related to ‘Mobilome: prophages, transposons (X)’ are quite abundant in the *N*. *seriolae* UTF1. The *N*. *seriolae* UTF1 genome contains 406 genes in this category, compared to only 71 in *N*. *farcinica* IFM 10152, 44 in *N*. *brasiliensis* HUJEG-1, 45 in *N*. *cyriacigeorgica* GUH-2 and 56 in *N*. *nova* SH22a (S4 Table). According to the comparative genomic map in Fig 1, these mobile element genes are interspersed throughout the *N*. *seriolae* UTF1genome, and their sequences correspond to the variable regions (Fig 1). Overall, the abundance of the genes related to mobile element proteins and unknown function in the *N*. *seriolae* UTF1 genome can partially explain the divergence of their genome structure and gene content from the other *Nocardia* genomes (Figs 1 and 3).

**Fig 6:**
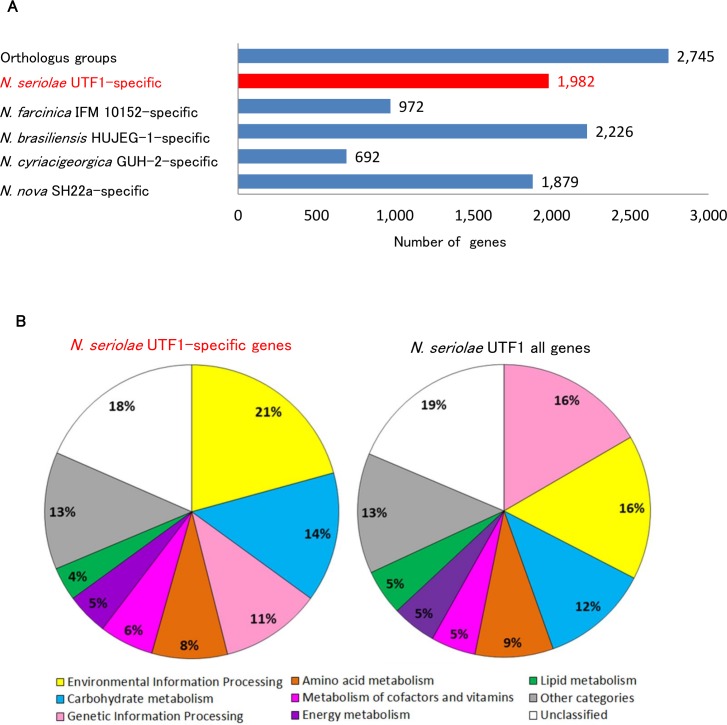
**Identification of *N*. *seriolae* UTF1-specific genes (A) and their functional annotations (B).** The orthologous and species-specific genes were identified using OrthoMCL [34] (A). The protein sequences of 1,982 *N*. *seriolae* UTF1-specific genes were functionally annotated with metabolic information from the Kyoto Encyclopedia of Genes and Genomes (KEGG) pathway database using KEGG Orthology And Links Annotation (BlastKOALA) program [27] (B).

To further characterize the genomic features of *N*. *seriolae* UTF1, we focus on the functional characterization of the *N*. *seriolae* UTF1-specific genes. As based on the OrthoMCL clustering, 2,745 orthologous genes were presented in all 5 *Nocardia* genomes and 1,982 genes were unique to *N*. *seriolae* UTF1 (Fig 6A). Of the 1,982 *N*. *seriolae* UTF1-specific genes, 217 genes (10.9%) were annotated in the KEGG database. The proportion of KEGG categories of the *N*. *seriolae* UTF1-specific genes shows some differences compared to those of all *N*. *seriolae* UTF1 genes (Fig 6B). The 'Environmental Information Processing' category (21%) are the most abundant in the *N*. *seriolae* UTF1-specific genes, followed by ‘Carbohydrate metabolism’ (14%) (Fig 6B). Focusing on the abundant KEGG modules, 14 genes are assigned in the ABC transport system (Module ID: M00254 and M00258) (Table 6). Because of limited resources in ocean environments, marine bacteria require various efficient transport systems to capture essential nutrients [77]. Therefore, the *N*. *seriolae* UTF1-specific transport systems-related genes identified in the present study may have a role in adaptation to the marine environment. In addition, most of the *N*. *seriolae* UTF1-specific genes (89.1%) were not assignable to KEGG. Further study on such transport systems-related genes and to the genes of unknown function will provide unique insight into their adaptation to fish hosts in the aquatic environment.

**Table 6:** Functional classification of unique genes of *N*. *seriolae* UTF1 by KEGG modules.

KEGG Module	Module ID	Description	Number of genes
**Environmental information processing**			
ABC-2 type and other transport systems	M00254	ABC-2 type transport system	12
	M00258	Putative ABC transport system	2
Mineral and organic ion transport system	M00188	NitT/TauT family transport system	1
	M00190	Iron(III) transport system	2
	M00299	Spermidine/putrescine transport system	4
Phosphotransferase system (PTS)	M00273	PTS system, fructose-specific II component	3
Phosphate and amino acid transport system	M00233	Glutamate transport system	1
Two-component regulatory system	M00452	CusS-CusR (copper tolerance) two-component regulatory system	2
	M00475	BarA-UvrY (central carbon metabolism) two-component regulatory system	1
Bacterial secretion system	M00335	Sec (secretion) system	1
Drug efflux transporter/pump	M00713	Fluoroquinolone resistance, efflux pump LfrA	1
Drug resistance	M00742	Aminoglycoside resistance, protease FtsH	1
	M00743	Aminoglycoside resistance, protease HtpX	1
	M00727	Cationic antimicrobial peptide (CAMP) resistance, N-acetylmuramoyl-L-alanine amidase AmiA and AmiC	1
	M00745	Imipenem resistance, repression of porin OprD	2
	M00714	Multidrug resistance, efflux pump QacA	1
**Energy metabolism**			
ATP synthesis	M00159	V-type ATPase, prokaryotes	5
	M00149	Succinate dehydrogenase, prokaryotes	4
	M00151	Cytochrome bc1 complex respiratory unit	1
Nitrogen metabolism	M00530	Dissimilatory nitrate reduction, nitrate = > ammonia	1
Methane metabolism	M00345	Formaldehyde assimilation, ribulose monophosphate pathway	1
**Carbohydrate and lipid metabolism**			
Fatty acid metabolism	M00083	Fatty acid biosynthesis, elongation	3
	M00082	Fatty acid biosynthesis, initiation	1
Central carbohydrate metabolism	M00307	Pyruvate oxidation, pyruvate = > acetyl-CoA	3
**Nucleotide and amino acid metabolism**			
Serine and threonine metabolism	M00555	Betaine biosynthesis, choline = > betaine	1
Cysteine and methionine metabolism	M00021	Cysteine biosynthesis, serine = > cysteine	2
Other amino acid metabolism	M00027	GABA (gamma-Aminobutyrate) shunt	1
**Genetic information processing**			
Ribosome	M00178	Ribosome, bacteria	1

## Conclusions

The complete nucleotide sequence of *N*. *seriolae* UTF1 consists of a circular chromosome of 8,121,733 bp with a G+C content of 68.1% and 7,697 predicted CDSs. The genome possesses known bacterial virulence genes that have functions in host cell invasion, modulation of phagocyte function and for survival within macrophages. The detected candidate virulence factors provide a novel resource for further study of their pathogenic mechanisms at the molecular level in the fish nocardiosis research community. We also found many antibiotic resistance genes on the *N*. *seriolae* UTF1 chromosome, suggesting natural resistance of this bacteria to many drugs. Our comparative analysis with the four existing complete *Nocardia* spp. genomes revealed that the *N*. *seriolae* UTF1 genome structure and gene content differs from the other *Nocardia* genomes due to a large amount of mobile element genes. In addition, there are homologs of many transporters among the *N*. *seriolae* UTF1-specific genes allowing us to speculate on their role in adaptation in the marine environment. Thus, we expect that the availability of the complete genome of *N*. *seriolae* UTF1 can be used as the reference sequence not only for *N*. *seriolae* isolates, but also for the comparative genomic studies of genus *Nocardia* as an example of a marine fish pathogen to provide insights into the ecological and functional diversity of this genus [1] in the near future.

## Supporting information

S1 Fig Identification and characterization of gap regions in the previous reported two *N*. *seriolae* draft genome sequences. The contig sequences of *N*. *seriolae* ZJ0503 (GenBank: NZ_JNCT01000000, 319 contigs) and N-2927 (GenBank: NZ_BAWD02000000, 339 contigs) were aligned to the complete sequence of *N*. *seriolae* UTF1 genome. Three hundred (average length: 1373 bp) and 297 (average length: 1502 bp) uncovered regions were detected in the comparison with ZJ0503 and with N-2927, respectively. These gap sequences were subjected to BLASTX search against the NCBI RefSeq database (*E* value threshold of 1*E*−5). As a result, 294 (98.0%) for ZJ0503 and 290 (97.6%) for N-2927 have significant BLAST hit. Values represent number of genes with the best BLAST hit for ZJ0503 (red) and N-2927 (blue).
(PDF)

S2 Fig Comparison of *N*. *seriolae* UTF1 genome and two other *N*. *seriolae* draft genomes, ZJ0503 (GenBank: NZ_JNCT01000000) and N-2927 (GenBank: NZ_BAWD02000000). The BLASTN-based ring image was generated by BLAST Ring Image Generator (BRIG) version 0.95 [32]. The innermost two rings show GC content (black) and GC skew (purple). The second and third innermost rings represent a BLASTN comparison with the draft genomes of ZJ0503 (blue) and N-2927 (red), respectively. Bars indicate the position of mobile-element related genes in the *N*. *seriolae* UTF1 genome such as transposases (black), endonuclease DDE (blue) and integrase (green).
(PDF)

S3 Fig Dot plot analysis of the genome sequence of four *Nocardia* species (*N*. *farcinica* IFM 10152, *N*. *brasiliensis* HUJEG-1, *N*. *cyriacigeorgica* GUH-2 and *N*. *nova* SH22a. Nucleotide-based alignments were performed with MUMmer version 3.22 and dot plots were generated by the mummerplot script and the Unix program gnuplot [31].
(PDF)

S4 Fig Functional profiling of the five *Nocardia* complete genomes with the dataset of overall subsystems. (TIF)

S5 Fig Functional profiling of the five *Nocardia* complete genomes with the KEGG modules assigned by BlastKOALA. (TIF)

S1 Table Long-range PCR primers used in this study. (XLSX)

S2 Table Candidate virulence factors identified in the *N*. *seriolae* UTF1 according to VFDB. (XLSX)

S3 Table Pairwise amino acid sequence similarities (%) of *N*. *seriolae* UTF1 Mce1 proteins with the other four *Nocardia* species, *Rhodococcus equi* and *Mycobacterium tuberculosis*. (XLSX)

S4 Table Comparison of COG distribution of *N*. *seriolae* UTF1 and the other four *Nocardia* genomes. (XLSX)
