# Supplementary material for: Analysis of the complete genome sequence of Nocardia seriolae UTF1, the causative agent of fish nocardiosis: The first reference genome sequence of the fish pathogenic Nocardia species
Source: PLoS One. 2017 Mar 3;12(3):e0173198. doi: 10.1371/journal.pone.0173198 (PMC5336288; doi:10.1371/journal.pone.0173198)
Supplement: S1 Fig — The contig sequences of N. seriolae ZJ0503 (GenBank: NZ_JNCT01000000, 319 contigs) and N-2927 (GenBank: NZ_BAWD02000000, 339 contigs) were aligned to the complete sequence of N. seriolae UTF1 genome. Three hundred (average length: 1373 bp) and 297 (average length: 1502 bp) uncovered regions were detected in the comparison with ZJ0503 and with N-2927, respectively. These gap sequences were subjected to BLASTX search against the NCBI RefSeq database (E value threshold of 1E−5). As a result, 294 (98.0%) for ZJ0503 and 290 (97.6%) for N-2927 have significant BLAST hit. Values represent number of genes with the best BLAST hit for ZJ0503 (red) and N-2927 (blue). (PDF) [file pone.0173198.s001.pdf]

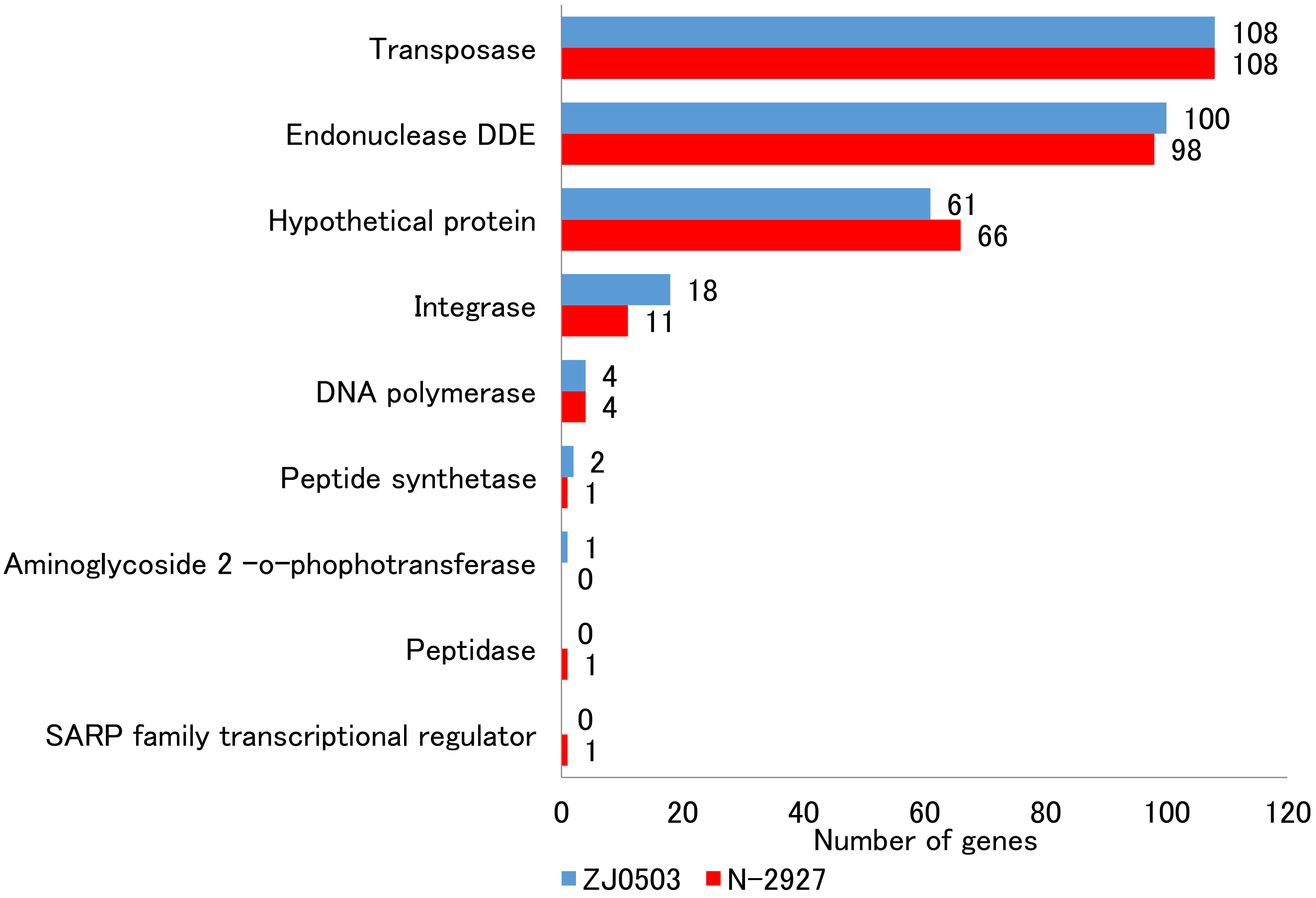

Figure S1. Identification and characterization of gap regions in the previous reported *N. seriolae* draft genome sequences. The contig sequences of *N. seriolae* ZJ0503 (gi: 696559939, 319 contigs) and N-2927 (gi: 780022598, 339 contigs) were aligned to the complete sequence of *N. seriolae* UTF1 genome. Three hundred (average length: 1373 bp) and 297 (average length: 1502 bp) uncovered regions were detected in the comparison with ZJ0503 and with N-2927, respectively. These gap sequences were subjected to BLASTX search against the NCBI RefSeq database (*E* value threshold of 1*E*–5). As a result, 294 (98.0%) for ZJ0503 and 290 (97.6%) for N-2927 have significant BLAST hit. Values represent number of genes with the best BLAST hit for ZJ0503 (red) and N-2927 (blue).
