# Supplementary material for: Analysis of the complete genome sequence of Nocardia seriolae UTF1, the causative agent of fish nocardiosis: The first reference genome sequence of the fish pathogenic Nocardia species
Source: PLoS One. 2017 Mar 3;12(3):e0173198. doi: 10.1371/journal.pone.0173198 (PMC5336288; doi:10.1371/journal.pone.0173198)
Supplement: S2 Fig — The BLASTN-based ring image was generated by BLAST Ring Image Generator (BRIG) version 0.95 [32]. The innermost two rings show GC content (black) and GC skew (purple). The second and third innermost rings represent a BLASTN comparison with the draft genomes of ZJ0503 (blue) and N-2927 (red), respectively. Bars indicate the position of mobile-element related genes in the N. seriolae UTF1 genome such as transposases (black), endonuclease DDE (blue) and integrase (green). (PDF) [file pone.0173198.s002.pdf]

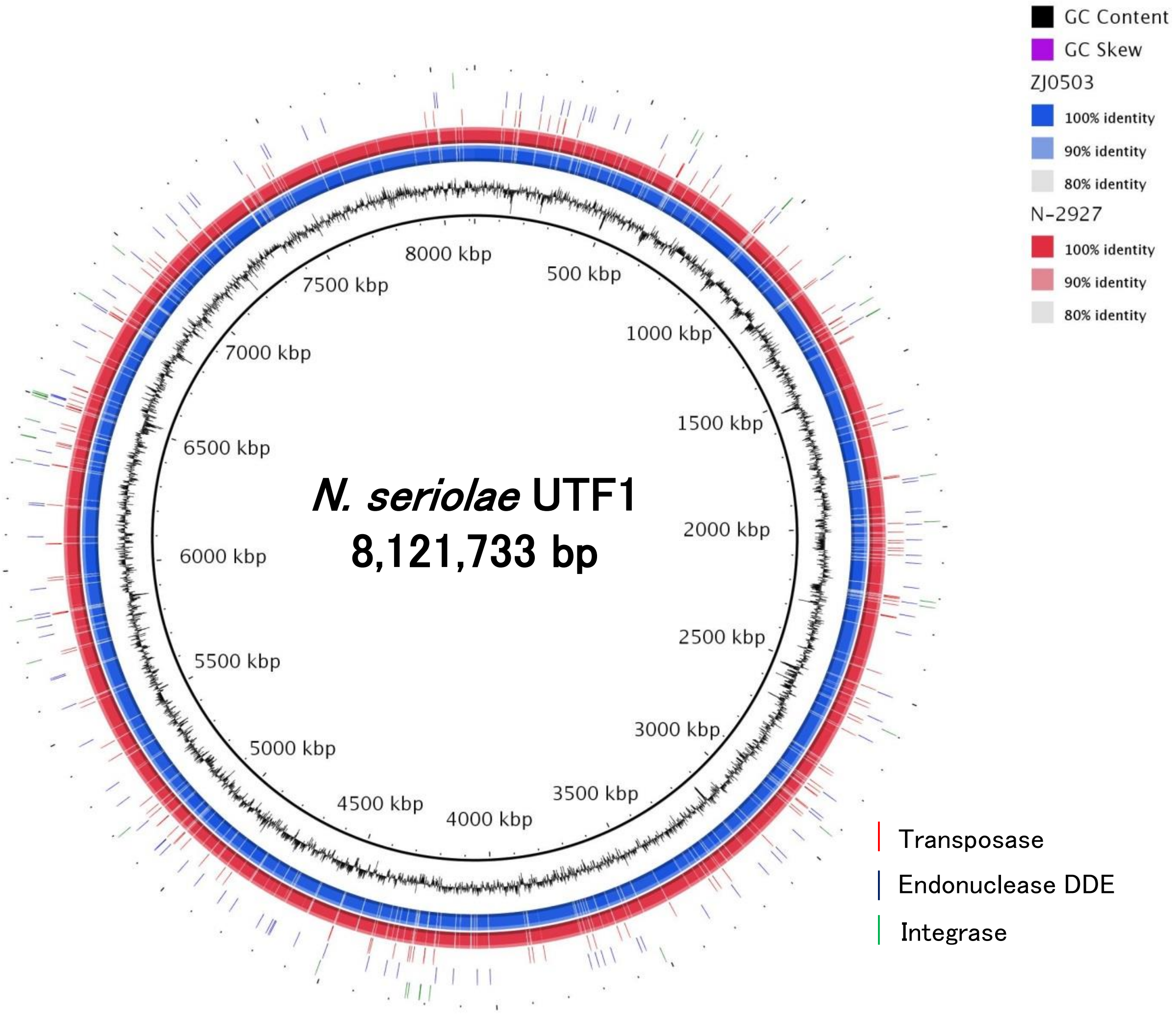


**Figure S2. Comparison of *N. seriolae* UTF1 genome and two other *N. seriolae* draft genomes, ZJ0503 (gi: 696559939) and N-2927 (gi: 780022598).** The BLASTN-based ring image was generated by BLAST Ring Image Generator (BRIG) version 0.95 [32]. The innermost two rings show GC content (black) and GC skew (purple). The second and third innermost rings represent a BLASTN comparison with draft genome of ZJ0503 (blue) and N-2927 (red). Bars indicate the position of mobile-element related genes in the *N. seriolae* UTF1 genome such as transposases (black), endonuclease DDE (blue) and integrase (green).
