# Supplementary material for: Analysis of the complete genome sequence of Nocardia seriolae UTF1, the causative agent of fish nocardiosis: The first reference genome sequence of the fish pathogenic Nocardia species
Source: PLoS One. 2017 Mar 3;12(3):e0173198. doi: 10.1371/journal.pone.0173198 (PMC5336288; doi:10.1371/journal.pone.0173198)
Supplement: S3 Fig — Nucleotide-based alignments were performed with MUMmer version 3.22 and dot plots were generated by the mummerplot script and the Unix program gnuplot [31]. (PDF) [file pone.0173198.s003.pdf]

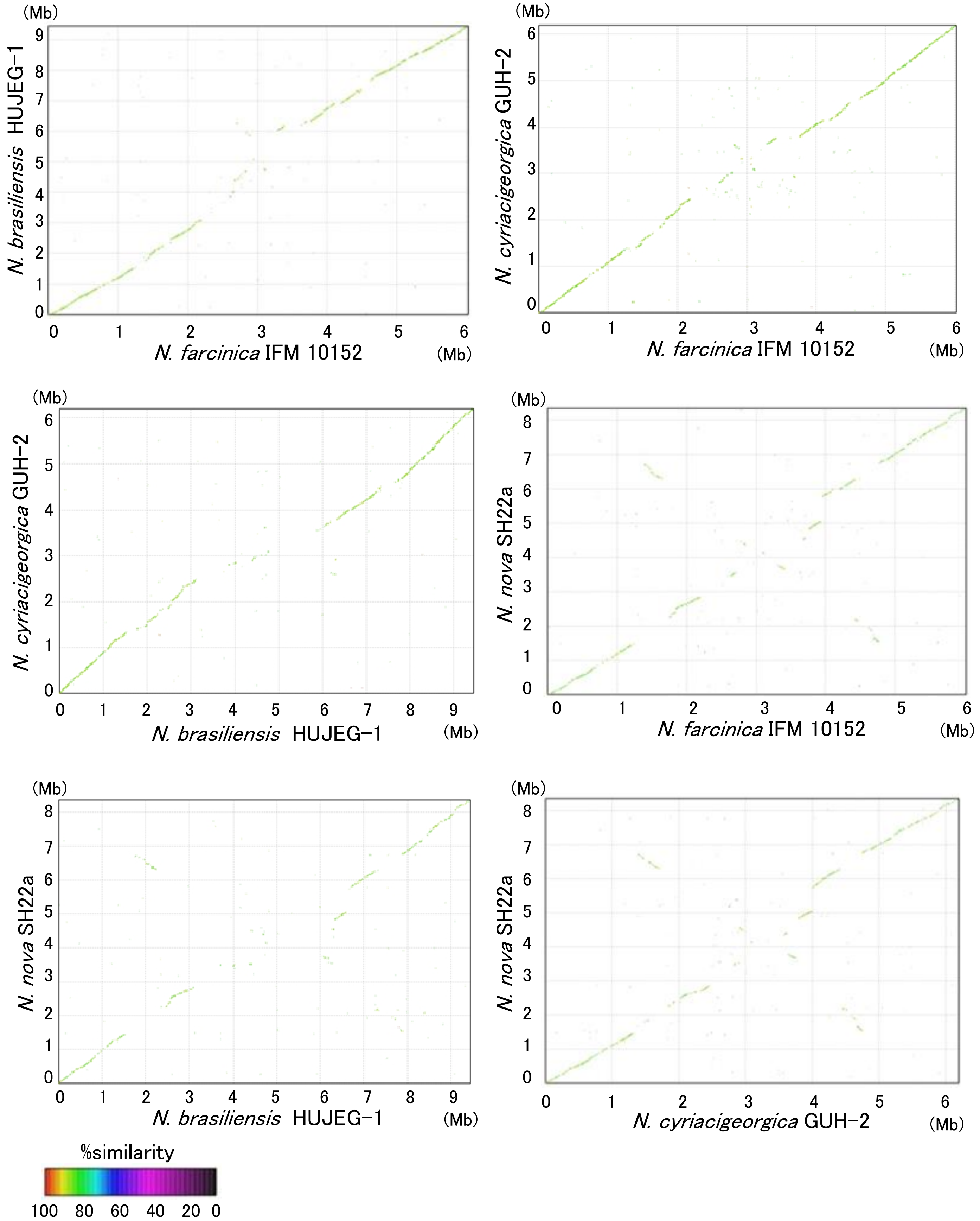

Figure S3. Dot plot analysis of the genome sequence of four *Nocardia* species (*N. farcinica* IFM 10152 , *N. brasiliensis* HUJEG-1, *N. cyriacigeorgica* GUH-2 and *N. nova* SH22a. Nucleotide-based alignments were performed with MUMmer version 3.22 and dot plots were generated by the mummerplot script and the Unix program gnuplot [31].
