# Supplementary figures and images for: Analysis of the complete genome sequence of Nocardia seriolae UTF1, the causative agent of fish nocardiosis: The first reference genome sequence of the fish pathogenic Nocardia species
Source: PLoS One. 2017 Mar 3;12(3):e0173198. doi: 10.1371/journal.pone.0173198 (PMC5336288; doi:10.1371/journal.pone.0173198)

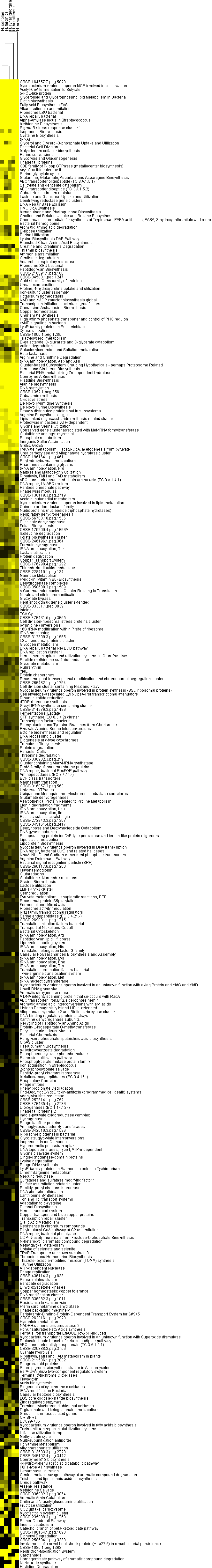

Supplement: S4 Fig — (TIF) [file pone.0173198.s004.tif]

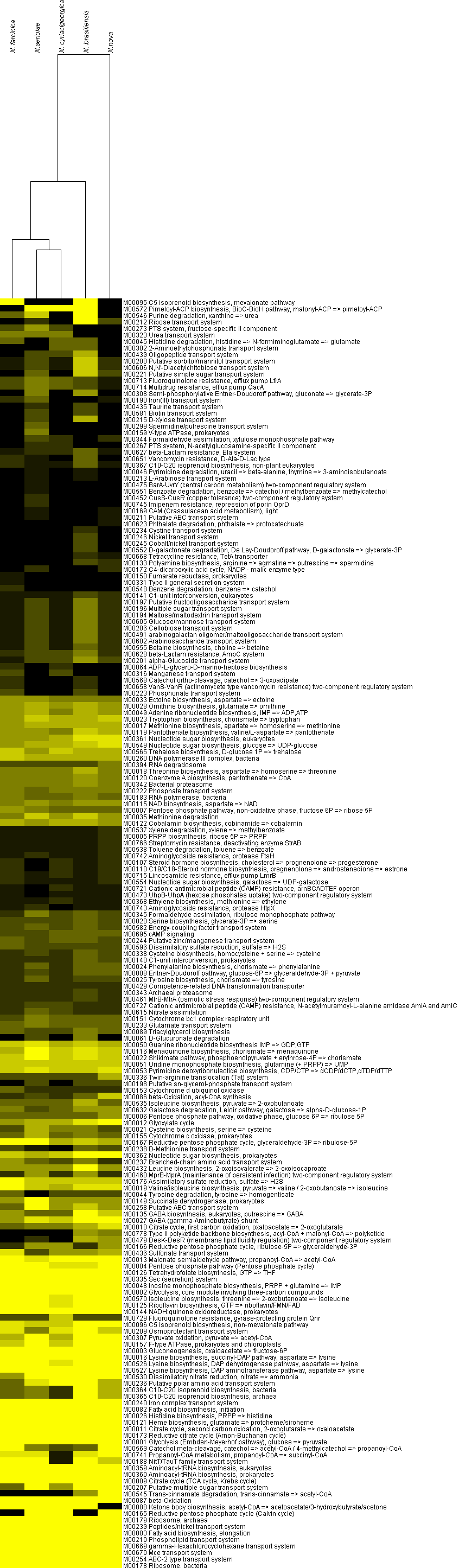

Supplement: S5 Fig — (TIF) [file pone.0173198.s005.tif]
